# Genome-Wide Survey of Invertase Encoding Genes and Functional Characterization of an Extracellular Fungal Pathogen-Responsive Invertase in *Glycine max*

**DOI:** 10.3390/ijms19082395

**Published:** 2018-08-14

**Authors:** Tao Su, Mei Han, Jie Min, Peixian Chen, Yuxin Mao, Qiao Huang, Qian Tong, Qiuchen Liu, Yanming Fang

**Affiliations:** Co-Innovation Center for Sustainable Forestry in Southern China, College of Biology and the Environment, Nanjing Forestry University, Nanjing 210037, China; sthanmei@njfu.edu.cn (M.H.); mj121451423@outlook.com (J.M.); peixian1998@163.com (P.C.); maoyuxina@outlook.com (Y.M.); 15977126951@163.com (Q.H.); tacy1210@outlook.com (Q.T.); qiuqiu980815@163.com (Q.L.); jwu4@njfu.edu.cn (Y.F.)

**Keywords:** invertase, source and sink, pathogen, drought tolerance, ABA, *Glycine max*

## Abstract

Invertases are essential enzymes that irreversibly catalyze the cleavage of sucrose into glucose and fructose. Cell wall invertase (CWI) and vacuolar invertase (VI) are glycosylated proteins and exert fundamental roles in plant growth as well as in response to environmental cues. As yet, comprehensive insight into invertase encoding genes are lacking in *Glycine max*. In the present study, the systematic survey of gene structures, coding regions, regulatory elements, conserved motifs, and phylogenies resulted in the identification of thirty–two putative invertase genes in soybean genome. Concomitantly, impacts on gene expression, enzyme activities, proteins, and soluble sugar accumulation were explored in specific tissues upon stress perturbation. In combination with the observation of subcellular compartmentation of the fluorescent fusion protein that indeed exported to apoplast, heterologous expression, and purification in using *Pichia pastoris* system revealed that GmCWI4 was a typical extracellular invertase. We postulated that GmCWI4 may play regulatory roles and be involved in pathogenic fungi defense. The experimental evaluation of physiological significance via phenotypic analysis of mutants under stress exposure has been initiated. Moreover, our paper provides theoretical basis for elucidating molecular mechanisms of invertase in association with inhibitors underlying the stress regime, and will contribute to the improvement of plant performance to a diverse range of stressors.

## 1. Introduction

In higher plants, sucrose is the major form of transported sugar, translocating from photosynthetic source leaves into non-photosynthetic sink tissues in higher plants [1]. The efficient use of sucrose as a core source of carbon and energy depends on its high cleavage capacity to produce hexoses (glucose and fructose). This process is mediated by two different key enzymes: sucrose synthase (Susy, EC 2.4.1.13) and invertase (EC 3.2.1.26). In the presence of Uridine Diphosphate (UDP), Susy converts sucrose into UDP-glucose and fructose, which are used for the cell respiration, biosynthesis of cell wall and starch, and the maintenance of sink strength [2]. In contrast to Susy, invertase irreversibly transforms sucrose into glucose and fructose, feeding plants with basic nutrients, and essential elements for sugar metabolism and signaling [3].

Based on the properties of the solubility, compartmentation, and pH-optimum, invertase is classified into two large subfamilies, cytoplasmic alkaline/neutral invertase (CI) and acid invertase (AI); the later contains cell wall invertase (CWI) and vacuolar invertase (VI). CI and AI are substantially distinct in molecular and biochemical characteristics. VI is soluble vacuole-localized enzyme with an optimal pH of 5.0–5.5, whereas CWI is insoluble, a cell wall bound enzyme with an optimal pH of 3.5–5.0, which is targeting to the apoplast. CWI and VI share similar conserved amino acid residues and are categorized into the glycoside hydrolase family 32 (GH32). Unlike the AI subfamily, CIs belong to the GH100 with an optimal pH of 6.8–9.0 and exhibit little homology to CWI and VI [4]. Besides, CI is a soluble enzyme and generally localized to the cytosol, mitochondria, plastids, and nucleus [5].

CWI and VI play multifaceted roles in source-sink interactions and responses to environmental cues via sugar metabolism and signaling pathway [6]. Apoplast-localized (extracellular) CWI converts sucrose into hexoses, which are further translocated from the phloem into apoplastic sink cells by sugar transporters. The unloaded hexoses may flux into intracellular compartments for metabolism and gene regulation, or into extracellular compartments for stress-related responses [7]. A maize CWI-deficient mutant, *Mn1,* led to reduced kernel size, which is a result of restrained endosperm development, whereas its overexpressing lines improved kernel weight and starch accumulation [8,9]. Moreover, fundamental roles of CWIs that are associated with seed development have been attempted in rice, tomato, and cotton [10,11]. In addition, CWIs were demonstrated to play critical roles in pathogen defense and abiotic stress tolerance [12,13,14]. Interestingly, more recent studies implicated potentials of CWIs in correlation with delay of the leaf aging [15,16]. Like CWI, roles of VI involved in carbon metabolism, hexose distribution, cellular osmolarity, and Reactive Oxygen Species (ROS) scavenging have been well documented in a variety of species [17,18]. Silencing of VI gene expression altered soluble sugar conversion, thereby reduced the cold-induced sweetening (CIS) in potato tubers [19,20]. Owing to technique difficulties in protein purification, enzymatic properties of CIs and their functions in vitro have not been well explored. Previously, CIs were postulated to liberate hexoses for tissues as compensate when the activities of AIs and Susy were low or insufficient [21]. Some reports revealed hitherto that CIs serve as a key player in regulating root growth [22], photosynthesis efficiency and nitrogen utilization [23], and ROS homeostasis [24]. A more recent study showed that a CI gene, *HbNIN2* of para rubber tree, influenced on sucrose catabolism and the latex accumulation [25]. In addition, suppression of CI activity resulted in altered cell wall production in hybrid aspen [26], prompting its central role in cellulose biosynthesis and carbon allocation.

CWI and VI are transcriptionally or posttranscriptionally regulated by diverse stress factors and phytohormone cues. Nevertheless, the regulation of CWI and VI activities has recently been experimentally verified to largely rely on the posttranslational control, which was intermediated by the cell wall or vacuolar inhibitor of β-fructosidases (C/VIFs). C/VIFs also play key roles on the inhibition of CWI and VI during seed and fruit development [15,27,28,29]. Suppression of VI activities by overexpressing VIF reduced the CIS and improved the processing qualities of potato [30,31]. However, the implications of CIFs in modulating the processes of apoplast-adapted stresses remain to be deciphered, particularly in plant immune responses to pathogen infections [32].

It has long been known that invertase exerts principal roles in maintaining hexose homeostasis for plant development and growth. To date, very few genes encoding for invertase were reported as well as manipulated extensive analyses of invertase candidates in *Glycine max*, which is a common crop. Here, invertase encoding genes were investigated by a genome-wide analysis within the latest genome annotation. Expression patterns of invertase in tissues and in response to stresses were thoroughly explored. We demonstrated that the possible links between gene expression patterns, proteins, enzyme activities, and sugar metabolites, prompting the modulation of CWI effectively incorporated into the process of multiple stress responses. Furthermore, *GmCWI4*, a fungal pathogen responsive gene, was experimentally characterized to be a typical extracellular invertase via analyses of its subcellular target and activities of recombinant protein in vitro. Overall, our findings provided a foundation for future in-depth elucidation of the physiological role in the regulation of sucrose metabolic processes and stress tolerance.

## 2. Results

### 2.1. Identification and Sequence Analyses of Soybean Invertase Candidates

A number of invertase isogenes of three subgroups have been characterized in some common plant species (Table 1). To identify invertase genes in soybean plant, systematic BLAST searches using known invertase sequences from Arabidopsis and rice as queries were conducted against the soybean genome. After removal of the redundant sequences, total of 32 putative invertase genes were identified, including 19 members (twelve *CWIs* and seven *VIs*) of AI subfamily and thirteen members of CI subfamily (Table S1). After manual reannotation, twelve CWI genes were designated *GmCWI1-12* based on identities with a previously reported *GmCWINV1* [33]. Seven VI genes and thirteen CI genes were named *GmVI1-7* and *GmCI1-13*, following the nomenclature that was proposed on the chromosome designation. Basic information of the gene ID, sizes and locations, coding sequence (CDS), and (open reading frame) ORF was presented. Interestingly, BLAST searches of the newly available soybean invertase sequences in GenBank resulted in findings of two novel CWIs (*GmCWI5* and *GmCWI12*). Along with the theoretical molecular weight (MW) and isoelectronic points (pI), in silico prediction of the subcellular target has also been performed (Table S1). Deduced invertase proteins contain 515–680 amino acid residues, and their MW range from 61.37 to 77 kDa, similar to the invertase in Arabidopsis and rice. In addition, proteins that were encoded by VI and CI genes hold acid pI ranging from 5.10 to 6.79, whereas most of the CWI proteins have the basic pI, which is a typical feature for the extracellular invertase.

### 2.2. Gene Structures and Cis-Elements

**Figure 1:**
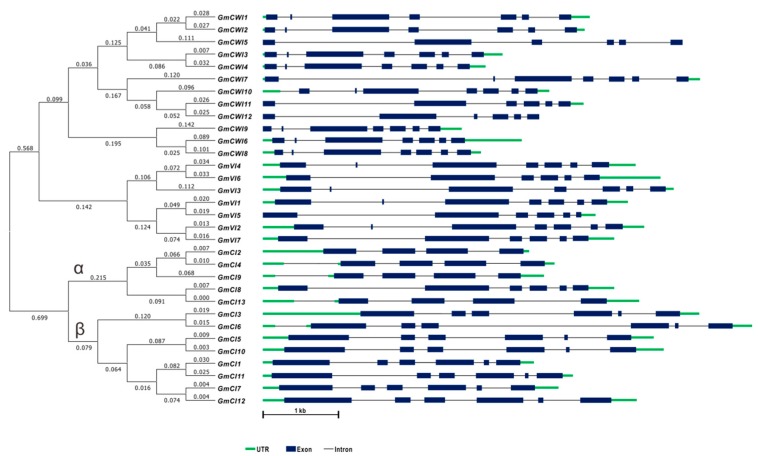
Cluster of soybean invertase gene candidates and their schematic gene structures. Exons and untranslated region (UTR) are indicated by dark blue boxes and green lines, respectively. Introns are represented in black lines. CIs were separated into α clade and β clade according to conserved amino acid sequences.

**Figure 2:**
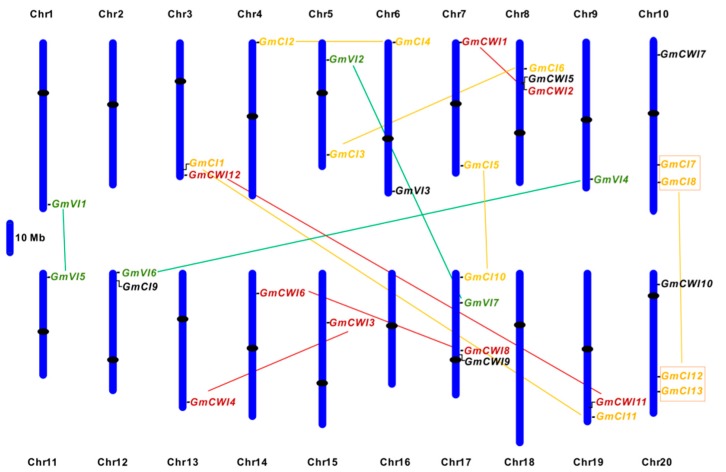
Invertase gene distributions in soybean chromosomes. Chromosomal distributions of invertase gene candidates are clustered on the basis of their physical locations on chromosome. Pairs of gene speculated to have undergone segmental duplication are positioned in same color and linked each other by a straight line. The centromeres are colored by black ovals.

To explore the possible genome organization and evolutionary relationship of soybean invertases, selected genes and their structures were analyzed. Searches among the soybean genome have retrieved thirty-two invertase-like genes that were shown to distribute among 17 of 20 soybean chromosomes; however, no invertase genes were found on chromosome 2, 16, and 18 (Table S1). Analyses of gene structures showed that *GmCWI* and *GmVI* generally contained six or seven exons and CI genes contained four or six exons, reflecting that these homologs are highly conserved in exon-intron patterns (Figure 1). Based on the gene physical locations, a chromosome map was generated, indicating that the gene density in each chromosome is uneven. As depicted in Figure 2, ten pairs of genes were predicted to cluster together and displayed high similarity (>90%) of amino acid sequences, suggesting the occurrence of the ancient genome duplication event, which may result in two copies of each gene undergone shuffling and rearrangement during genome evolution [42]. Most *GmVIs* and *GmCIs* were identified to be localized towards the chromosome ends. Two CWI genes, *GmCWI8* and *GmCWI9,* were localized on chromosome 17 and shared 78% identities in amino acid sequence. Both *GmCWI2* and *GmCWI5* were localized on chromosome 8, sharing 82% identities, implying that they might evolve as a result of tandem duplications.

**Figure 3:**
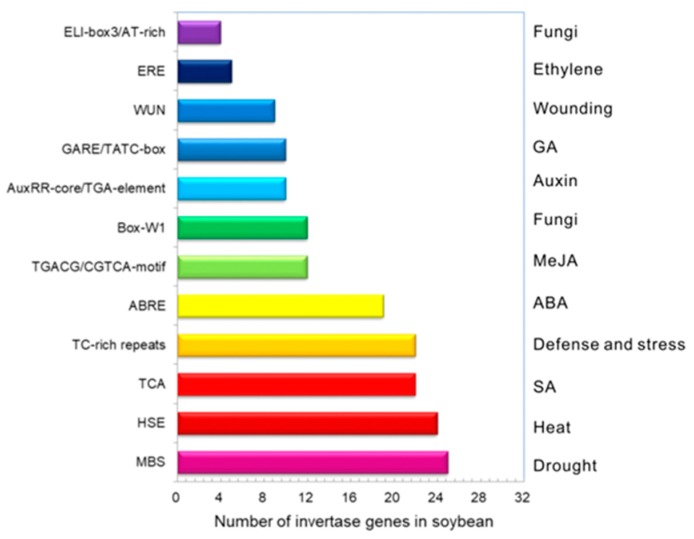
Prediction of the various cis-acting elements involved in environmental stresses and phytohormone responsiveness. ABRE (ABA-responsiveness), MBS (drought inducible MYB-binding site), TGACG/CGTCA-motif (MeJA-responsiveness), TCA-element (SA responsiveness), WUN-motif (wound-responsive element), TC-rich repeats (defense and stress responsiveness), Box-W1 (fungi elicitor responsiveness), ERE (ethylene-responsive element), ELI-box3/AT-rich (fungi elicitor responsiveness), AT-rich sequence (elicitor-mediated activation), TATC-box/GARE-motif (GA-responsiveness), AuxRR-core/TGA-element (auxin responsiveness), and HSE (heat responsiveness). Approximate 1.5 kb promoter of a specific gene was analysed by the online program PlantCARE (http://bioinformatics.psb.ugent.be/webtools/plantcare/html/) to detect the cis-regulatory elements.

Regulatory elements on gene promoters are the vital clues to identify the environmental factors that promote gene spatiotemporal expressions. To search the cis-acting elements in correlation with stresses and phytohormone responses, approximately 1500 bp upstream promoters of each invertase sequences were input in PlantCARE. It was shown that drought-responsive elements (MBS) were abundant in promoters of 25 invertase genes (Figure 3). SA-responsive elements (TCA) together with defense and stress-responsive elements (TC-rich repeats) were identified in the promoters of 22 invertase genes. More than 9 gene promoters contained WUN motifs that are involved in wounding-responsiveness. MeJA-responsive elements (TGACG/CGTCA-motifs) and fungus-related elements (Box-W1) were found in the promoters of 12 invertase genes, most of which were *GmCWI* and *GmVI*. In addition, eighteen invertase genes contained Abscisic acid (ABA)-responsive elements (ABRE), whereas a few of invertase genes contained cis-elements involved in ethylene (ERE), GA (GARE/TATC-box) and auxin (AuxRR-core/TGA-element) responses. Documentation of cis-elements suggested that genes might be up-and-down regulated by various environmental stimuli.

### 2.3. Conserved Domains and Phylogenetic Analyses

**Figure 4:**
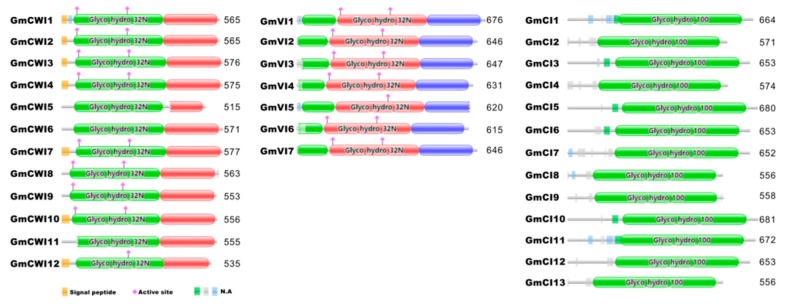
Deduction of conserved domains of the soybean invertase candidates. The diagram shows that cell wall invertase (CWI) and vacuolar invertase (VI) belong to glycoside hydrolase family 32 (GH32), whereas CI belongs to GH100. Numbers behind of each diagram indicate the number of amino acids. Predictions of the conserved domains were programed by using the PFAM (http://pfam.xfam.org/)

To gain insight into the conserved patterns, multiple sequences of the identified invertase candidates were aligned via Clustal Omega and concurrently, conserved domains were deduced using the PFAM (http://pfam.xfam.org). Previous reports revealed the typical features of 13 well-conserved functional regions for AI gene family, including two essential structures, β-fructosidase motif (NDPNG) and catalytic site (WECP/VDF), whereas 12 conserved motifs for CI [35]. With the exception of GmCWI6, 11, 12, and GmVI5 that are short of full β-fructosidase motif, all the members of CWI and VI contained well-conserved motifs, including WECP/VDF, the enzyme catalytic site (Figure 4 and Figure S1). In contrast, each member in CI subgroup retained 12 intact well-conserved regions, as indicated. Notably, a conserved amino acid residue (Threonine/Valine, T/V) from the ninth conserved motif was varied within the CI gene family (Figure S2). Based on the protein sequence identities, it was postulated that T/V confers substantial amino acid to divide CIs into α clade and β clade (Figure 1). Analyses of the deduced protein structures showed different conserved patterns between AI and CI subfamilies, prompted that they were logically grouped into different gene families, namely GH32 and GH100, respectively.

**Figure 5:**
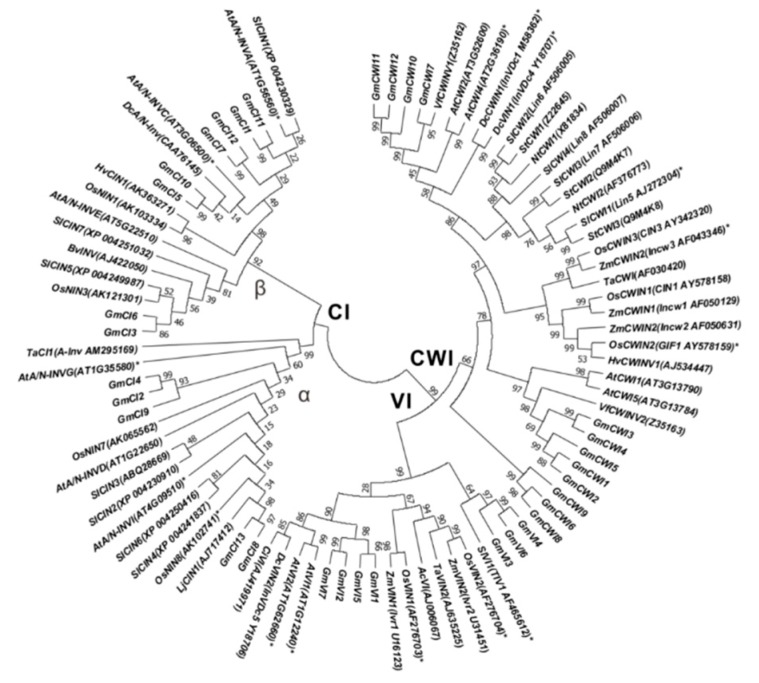
Phylogenetic relations of soybean invertase candidates with homologs from other plant species. Asterisks indicate genes that were functionally characterized by mutational studies. Phylogenetic tree was generated with Clustal alignment in MEGA 6 using neighbor-joining method. At, *Arabidopsis thaliana*; Dc, *Daucus carota*; Lj, *Lotus japonicus*; Os, *Oryza sativa*; Sl, *Solanum lycopersicum*; Vf, *Vicia faba*; Zm, *Zea mays*; Hv, *Hordeum vulgare*; Ta, *Triticum aestivum*; Bv, *Beta vulgaris*. The accession number and locus ID of each invertase homolog are provided in parentheses.

To assess the evolutionary history and distinct origin, multiple amino acid sequences of AI and CI subfamilies were aligned by using the neighbor-joining method gin MEGA 6.0 [43]. Alignment of full-length protein sequences and a comparison of the exon/intron organization, an unrooted phylogenetic tree was constructed (Figure S3). Thirty-two candidate invertases were categorized into three subgroups, whereas CI subgroup was evolutionarily far away from the CWI and VI subgroups. Further phylogenetic analyses with a large number of invertase homologs, including specific genes that have been experimentally verified by mutation studies in Arabidopsis, rice, carrot, corn, and tomato, suggested that these invertase homologs were highly conserved among various plant species (Figure 5). Interestingly, most members of *GmCIs* predicted targeting of cytosols or plasma membrane are clustered in α clade, whereas genes localized to mitochondria and chloroplasts are clustered in β clade, suggesting the possible correlation between phylogenetic relation and in silico subcellular localization within the CI subfamily (Figure 5). Besides, conserved amino acids of the enzyme catalytic site (WECP/VDF) were identified to be determinants to distinguish CWI clades from VI clades within the AI subfamily.

### 2.4. Transcriptome Profiling of Invertase Genes

**Figure 6:**
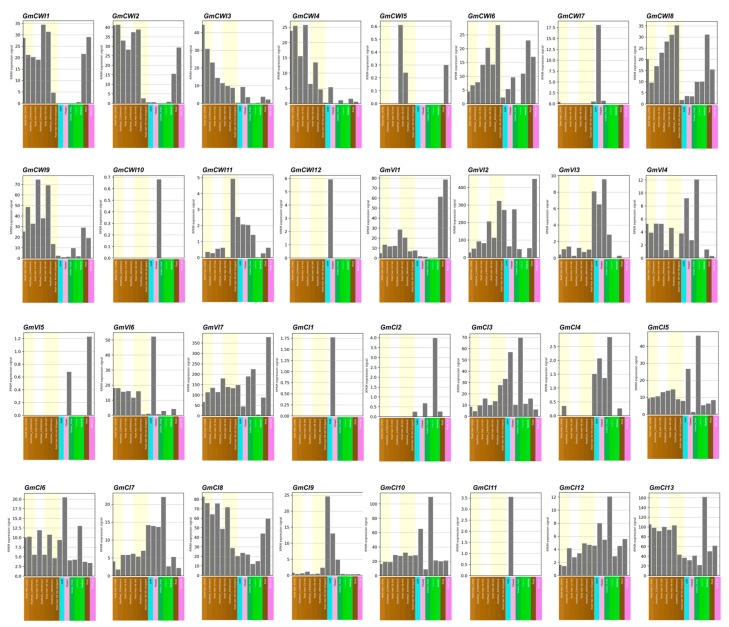
Transcriptomic expression patterns of invertase genes in different tissues of soybean. Tissue-specific expression was derived and analyzed by RNA-seq. Based on the available database of soybean eFP Browser, expression values are given in region per million mapped reads (RPKM) and all materials were sampled in triplicate.

A total of 32 soybean invertase genes were identified and deduced with well-conserved genomic (or protein) structures. To investigate transcriptomic profiles in sources and sinks, all of the putative invertase genes were initially investigated by the RNA-seq. Based on the available database of tissue-specific expression patterns from the Soybean eFP Browser (http://bar.utoronto.ca/efpsoybean/cgi-bin/efpWeb.cgi), it was found that *GmCWI7* and *GmCWI12* were specifically expressed in flower within CWI subgroup (Figure 6). Three members (*GmCWI8*, *9* and *11*) of CWI were predominantly expressed in leaves, and at least 6 CWI isogenes (*GmCWI1-4*, *6*, *8,* and *9*) showed high level of transcripts in roots. In addition, transcripts of *GmCWI5* and *GmCWI10* appeared to be lower than the detected level in all tissues. In contrast, RNA-seq analysis of VI subgroup revealed that *GmVI1* and *GmVI6* were dominantly expressed in roots and flowers, respectively. Three members (*GmVI2*, *4* and *7*) exhibited universal transcripts in every tissue, with particularly high degrees in both leaves and roots; however, almost no transcripts of *GmVI5* were detected in these tissues. Among CI subgroup, four members (*GmCI2*, *4*, *11,* and *12*) were shown to display very low levels of transcripts and *GmCI1* and *GmCI9* were specifically expressed in flowers (Figure 6), whereas the rest members of CI genes were found to be universally expressed. Notably, transcriptomic analyses of invertase candidates revealed the possible correlation between duplicated gene pairs, which showed similar transcriptomic patterns, particularly for the CI subgroup (e.g., *GmCI1/11*, *GmCI2/4*, *GmCI5/10*, and *GmCI8/13*).

### 2.5. Expression Validation in Tissues as well as in Response to Stress Stimulus

**Figure 7:**
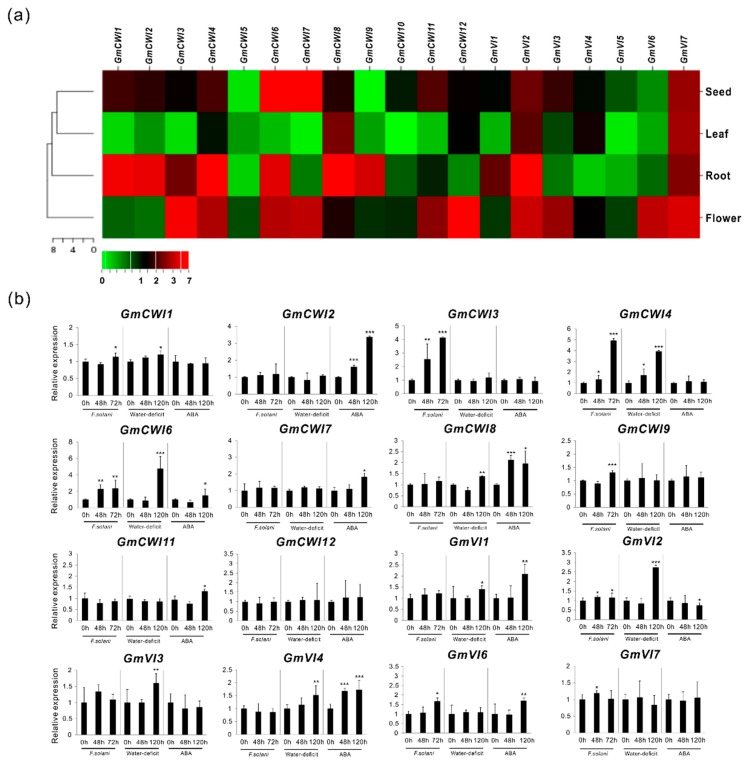
Expression validation of the acid invertase genes. Relative expressions in various tissues as well as in response to stress factors (*F. solani*, Water-deficit, and ABA) were conducted by qRT-PCR (**a**,**b**). Data represent mean value of at least three independent biological replicates. *GmACT2/7*, *GmACTII*, *GmEF/αb*, and *GmCYP* were used as reference genes. Asterisks indicate significant differences in comparison with the control using Student’s *t*-test: *p* < 0.001 (***), *p* < 0.01 (**), *p* < 0.05 (*).

To evaluate RNA-seq data and identify genes that were expressed in a specific tissue, CWI and VI were further explored their expressions by qRT-PCR in four selected tissues (roots, leaves, flowers, and seeds). As shown in heat map (Figure 7a), expression validation confirmed that most of the *GmCWI* transcripts were enriched in roots. Three members (*GmCWI4*, *8*, and *12*) were identified to be highly expressed in leaves. *GmCWI6* and *GmCWI7* showed much higher expression levels in seeds. However, no transcripts of *GmCWI5* and *GmCWI10* were detected in roots and leaves, which fit well with RNA-seq data (Figure 6). Within the VI subgroup, more members (*GmVI1*, *GmVI2*, *GmVI4,* and *GmVI7*) showed their transcript abundance in either roots or leaves, except for *GmVI5*, which was not technically detected its expression in all tissues. Nevertheless, inspection of expression patterns by qRT-PCR assay was mostly compatible with transcriptomic RNA-seq data, whereas only few discrepancies observed are probably due to the genetic variation.

Evaluation of gene expressions by qRT-PCR resulted in the identification of 10 *GmCWIs* and 6 *GmVIs*, showing detected transcripts in roots or leaves (Figure 7a). Previous reports revealed that CWIs function as defense genes during the course of plant-pathogen interaction [44]. Also, numerous studies uncovered that the transcripts of CWIs were significantly promoted in source tissues upon drought (or water-deficit) stress and ABA-induced leaf senescence [45,46]. Thus, to identify the biotic and abiotic stress-responsive genes, transcripts of *GmCWIs* and *GmVIs* in root infected by pathogenic *F. solani*, and in leaf under water-deficit as well as ABA perturbation, were explored thereafter. As shown in Figure 7b, after the inoculation of *F. solani* into roots for 48 h, transcripts of three CWI genes (*GmCWI3*, *GmCWI4,* and *GmCWI6*) and two VI genes (*GmVI2* and *GmVI7*) were found to be significantly induced. When inoculation time was extended to 72 h, *GmCWI9* was subsequently upregulated. *GmCWI3*, *GmCWI4,* and *GmVI6* revealed a drastic increase of their transcripts. Under abiotic stress conditions, transcript analyses during the time course (48 h and 120 h) of water-deficit depicted that three CWI genes (*GmCWI4*, *GmCWI6,* and *GmCWI8*) together with four VI genes (*GmVI1-GmVI4*) were constantly induced. By contrast, three CWI genes (*GmCWI2*, *GmCWI7,* and *GmCWI8*) were detected constant increases upon ABA treatment (Figure 7b). One VI genes, *GmVI4* was found a significant induction at 48 h. When the time was prolonged to 120 h, *GmVI1* and *GmVI6* showed increases of their transcripts. However, *GmVI2* appeared to be down-regulated in response to ABA perturbation.

An increasing number of studies showed that regulation of CWIs were also subject to posttranslational control that was accompanied by co-expression of inhibitory proteins [15,28,29,47]. This frequently occurred phenomenon allowed for us to explore whether CWIs were co-expressed with their inhibitors, transcripts of two functionally characterized inhibitor genes were quantified concurrently by qRT-PCR. Infection of *F. solani* caused a constant suppression of *GmC/VIF2*, whereas the expression of *GmCIF1* appeared to be slightly affected, indicating that *GmC/VIF2* is a fungal pathogen-responsive gene (Figure S4). Besides, both *GmCIF1* and *GmC/VIF2* were promoted in responses to water-deficit and ABA perturbation. Overall, no *GmCWIs* were likely co-expressed with *GmC/VIF2* during the pathogenic infection; however, more than three CWI genes showed their potential co-expression patterns with both *GmCIF1* and *GmC/VIF2* under conditions of the water-deficit and ABA (Figure 7b and Figure S4).

### 2.6. Effects of CWI and VI Activities, and Soluble Sugar Accumulation

**Figure 8:**
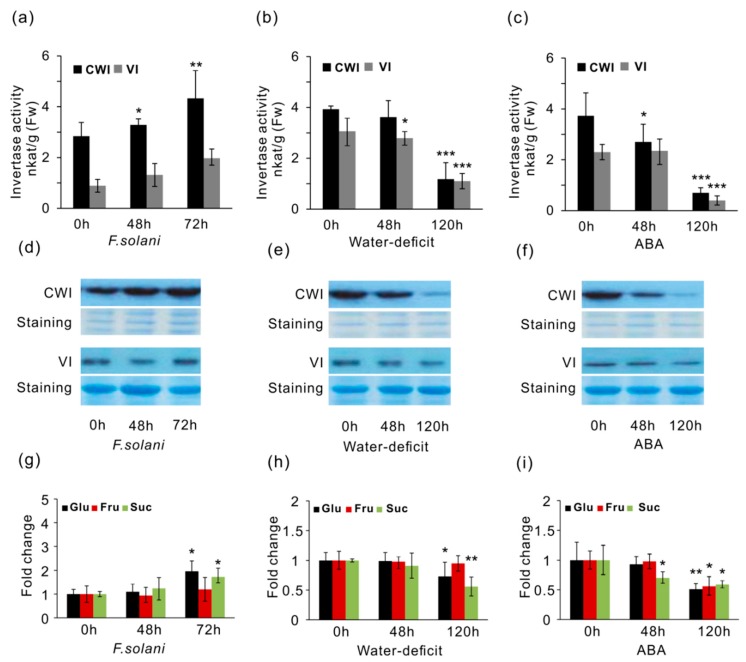
Effects of acid invertase activities, proteins, and soluble sugar accumulation. CWI and VI activities (**a**–**c**), proteins (**d**–**f**), and soluble sugar (glucose, fructose, and sucrose) content (**g**–**i**) were inspected in response to *F. solani*, water-deficit and ABA. Polyclonal antibodies of CWI and VI were generated from BvVI1 and BvCWI-1 in sugar beet. Data of enzyme activities represent means ±SE of at least four independent biological replicates. Data of sugar quantification represent mean ±SE of at least six biological replicates. Asterisks indicate significant differences in comparison with the control using Student’s *t*-test: *p* < 0.001 (***), *p* < 0.01 (**), *p* < 0.05 (*).

To determine the effects on enzyme activities in response to stress factors, CWI and VI were extracted and examined in vitro. Measurement of enzyme activities in different soybean tissues showed that CWI activities were more predominant than VI activities in mature leaves and roots, with exception in developing seeds and flowers (Figure S5). Under pathogenic stress of *F. solani*, CWI activities were slightly elevated by approximate 15% at 48 h (Figure 8a). After 72 h, CWI activities increased 35% than the control (0 h). However, VI activities were not significantly affected within the whole period of infection, suggesting that VI might not be involved in the fungal pathogen-induced defense response. In contrast, under water-deficit conditions, only VI activities showed statistic decreases at 48 h, and after water was withheld for 120 h, both CWI and VI were significantly repressed (Figure 8b). ABA significantly suppressed the activities of CWI and VI during the period of treatment, particularly when the time was extended to 120 h (Figure 8c). As both water-deficit and ABA can trigger leaf senescence, and therefore, it was not surprising that marked decreases of CWI and VI activities at 120 h were observed parallel in plants that were stressed with water-deficit and ABA. Moreover, under same stress conditions, western blotting analyses with CWI and VI antibodies derived from sugar beet implicated that CWI increased upon the infection of *F. solani*, whereas they decreased upon water-deficit and ABA perturbation (Figure 8d–f), which corroborated the detected changes of CWI activities in respective tissues.

Altered enzyme activities prompted us to examine whether the production of sugar metabolites were influenced. Under pathogen infection, we found that elevated CWI activities resulted in unchanged levels of glucose at 48 h. After longer time (72 h) of infection, both glucose and sucrose contents significantly increased (Figure 8g). However, after 120 h treatments, abiotic stresses of water-deficit and ABA led to a drastic reduction of glucose and sucrose (Figure 8h,i). Interestingly, fructose accumulation appeared to be not significantly affected upon *F. solani* and water-deficit (Figure 8g,h). Concurrent with a marked suppression of CWI activities, glucose found a significant reduction in response to water-deficit and ABA at 120 h, suggesting that the modified activities of CWI and VI were able to directly impact hexose pool within apoplastic spaces, particularly for the deeply stressed plants. 

### 2.7. Subcellular Compartmentation of GmCWI4

**Figure 9:**
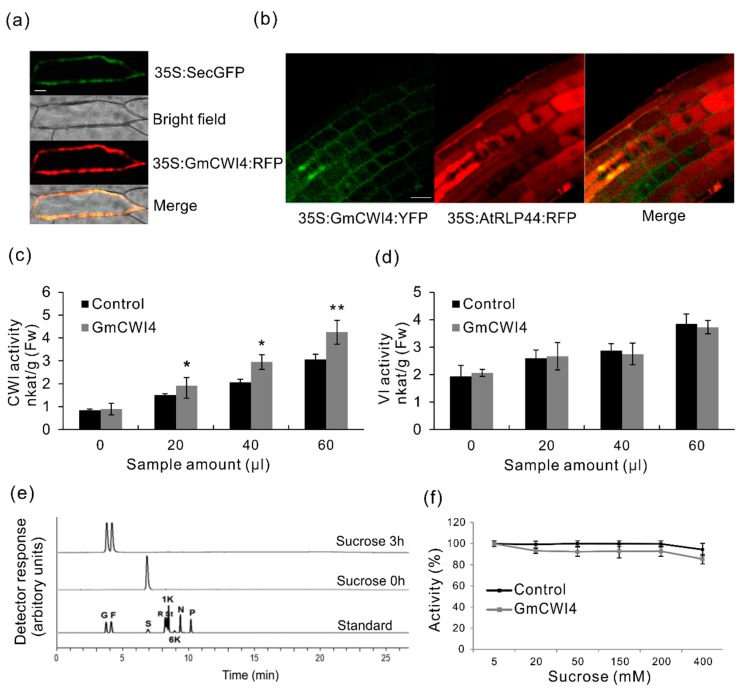
Detection of the subcellular localization and functional activities of GmCWI4 in vitro. Apoplastic targeting images of GmCWI4 red fluorescent protein/yellow fluorescent protein (RFP/YFP) fusion proteins were visualized in onion epidermis (**a**) and in transgenic Arabidopsis root tips (**b**) by confocal laser scanning microscopy (CLSM). Heterologous expression of GmCWI4 in tobacco leaves via infiltration showed extracted CWI activities (**c**) and VI activities (**d**). The infiltration buffer with *P19* plasmid was used as a control. Recombinant proteins were purified from *Pichia pastoris* via immobilized metal ion affinity chromatography on Ni-IDA resin under native conditions. The hydrolysis activities were measured with sucrose as substrate (**e**). Comparison of specificities of enzyme activities between the recombinant protein and the acid invertase via commercial purchase (**f**). The substrates (50 μL) were incubated with 50 μL of the recombinant protein (25 μg) for 0 and 3 h, and subsequently were analyzed by HPAEC-PAD chromatograms. Data are means of three biological replicates. Abbreviations for each sugar peak in the standard are: G, glucose; F, fructose; S, sucrose; R, raffinose; St, starchyose; 1K, 1-kestotriose; 6K, 6-kestotriose; N, 1,1-nystose; P, 1,1,1-kestopentaose. Data of enzyme activities represent means ±SE of at least four independent biological replicates. Asterisks indicate significant differences in comparison with the control using Student’s *t*-test: *p* < 0.01 (**), *p* < 0.05 (*).

Genome-wide survey showed that twelve CWI candidates were identified in soybean genome; however, neither of them has been reported with genuine sucrose hydrolysis activities in vitro as well as the subcellular localizations. The deduced GmCWI4 contains 574 amino acids, including 17 residues as putative signal peptides at N-terminus. Mature protein of GmCWI4 is 62.64 kDa, holding a basic pI of 9.17 (Table S1). Firstly, to examine the subcellular target, a GmCWI4-RFP fusion construct was bombarded into onion epidermis, and, alternatively, a GmCWI4-YFP fusion construct was introduced into Arabidopsis to create transgenic plants by floral dip. Both of the constructs were driven with the constitutive 35S promoter. For the former approach transformed in onion epidermal cells, an extracellular marker of secretory GFP (SecGFP) was used as a cell wall control, showing green fluorescence throughout the peripherals of cells (Figure 9a). By overlapping GFP signal, the same subcellular targeting patterns of GmCWI4-RFP were visualized, whereas no fluorescent signals were detected in vacuoles. Co-localization of fluorescent signals GFP and RFP in onion epidermal cells suggested the apoplastic targeting of GmCWI4. As shown in Figure 9b, further observation of YFP fluorescence of GmCWI4-YFP fusion construction in transgenic Arabidopsis root tips showed intense signals that were restricted in the cell wall, but a distinct targeting pattern from the plasma membrane marker, AtRLP44 [48]. Thus, both transient and stable expression assays suggested that GmCWI4 was predominantly localized to the apoplast, which was perfectly in line with the in silico prediction of its subcellular compartmentation (Table S1).

### 2.8. Functional Characterization of GmCWI4

To further examine whether *GmCWI4* functions as an invertase in vitro, we used a transient expression system in tobacco leaves in combination with the recombinant protein expression in the methylotrophic yeast *Pichia pastoris*. The latter was purified via an approach of affinity chromatography. As shown in Figure 9c, with an increasing amount of sample infiltrated in tobacco leaves, extracted CWI activities of one half leaves by overexpressing *P19* and *pMDC32-GmCWI4* plasmids increased more significantly than the control, another half leaves with P19 plasmid. However, VI activities appeared to be not apparently affected, showing similar levels to the control (Figure 9d), prompting that transiently overexpressing *GmCWI4* in tobacco leaves particularly altered CWI activities. Besides, to verify the enzymatic properties, a tagged construct of *pPICZαA-GmCWI4* was transformed into *Pichia pastoris* wild type strains (*X-33*). Cell culture supernatants were initially harvested, concentrated, and desalted after methanol (0.5%) induction (Figure S6a). Coomassie stained SDS-PAGE gel indicated that the recombinant GmCWI4 was perfectly purified by nickel affinity chromatography on Ni-IDA resin. Immunoblotting analysis with a c-Myc antibody confirmed that the recombinant protein largely accumulated in flow through (FT) fractions (Figure S6b). To detect the enzymatic specificity, an appropriate volume of purified protein was taken and the reaction was quantified by measuring liberated glucose and fructose from the hydrolysis of substrate sucrose with HPAEC-PAD. After three hours, productions of glucose and fructose were clearly observed from the substrate, suggesting that the activity elevated with increases of induction time (Figure 9e), whereas no fructan exohydrolase (FEH) activities were detected by using fructan-related standard (data not shown). Purified recombinant protein showed optimal enzyme activities at 35 °C in the acid buffer (pH = 6) (Figure S6c,d). Besides, with an increasing concentration of substrate, percentage of enzyme activities was found with similar patterns as control, a commercially purchased acid invertase (Figure 9f). To sum up, these data suggested that GmCWI4 is a typical extracellular enzyme, showing sucrose hydrolysis activities. However, further detailed work is required to validate and unveil the physiological function and regulatory roles of *GmCWI4* in the soybean plant.

## 3. Discussion

### 3.1. Invertase Gene Family in Soybean

Emerging reports revealed that CI played important actions in the regulation of plant normal growth and the CWI and VI exerted pivotal roles in sustaining sink strength and stress tolerance. The recent availability of soybean genome information allowed for us to deeply explore the different invertase gene families. However, over the past few years, a limited number of invertase has been functionally characterized in plants, particularly in soybean. To our knowledge, to date, only *GmCWINV1* (*GmCWI1*) has been isolated on the basis of its spatiotemporal expression [33]. Lacking of extensive analyses of invertase encoding genes and understanding of their genomic and expression properties has hampered unraveling their physiological roles as well as the regulatory mechanism behind them. In this study, through a genome-wide survey, thirty-two invertase genes were characterized in soybean. Based on the sequence and phylogenetic analyses, putative invertase genes were classified into three subgroups, displaying distinct gene structures and conserved protein features. Within CWI and VI subgroups, protein sequence alignment revealed that they shared a different identity ranging from 47.7% to 92.9%, and members in CI subgroup shared the identity more than 54% (Figure S3). Most members of CWI predicted to contain intact signal peptides at N terminal region and essential active sites, whereas all the members of VI subgroup featured the conserved catalytic site (WECVDF) (Figure 4 and Figure S1), and therefore, CWI and VI belong to GH32 family, whereas CI belongs to GH100 family [49]. Prediction of the subcellular localization by PSORT and CELLO implicated that members within different subgroups showed similar targeting properties (Table S1). Unfortunately, the in silico prediction of any members in VI subgroup failed to deduce the detailed subcellular targets (tonoplast or lumen). Thus, further experimental investigation is required to unravel the precise target and trafficking mechanisms for the GmVIs. In contrast to other plant species, including dicotyledonous angiosperms and the monocotyledonous angiosperm, actually more numbers of CWI and VI candidates were identified in soybean genome, reflecting that these candidates may exert more multifaceted actions during plant development and stress adaptation. However, early genetic studies suggested that soybean genome has evolved with several duplication events, which resulted in the outcome of some novel genes and functions [50]. Gene complexity and duplications may give rise to more challenges in evaluation and elucidation of the physiological roles of a unique invertase gene in soybean.

### 3.2. Mining Transcriptional Profiles of Invertase Genes

Tissue-specific expression pattern has been considered as one of the effective approaches to evaluate a candidate gene that is involved in a particular process of the plant development [51]. One strategy for unravelling how plants respond to stress stimuli is to analyze gene expression profiling while using transcription profiling technology [52]. As one of the initial steps, we used RNA-seq to examine the expression patterns of 32 invertase gene candidates in different soybean tissues. Transcriptomic analyses showed that CI genes appeared to be expressed spatially in all developmental stages, whereas most of the CWI and VI genes were expressed specifically in reproductive tissues, indicating that they may function at a particular stage of plant growth (Figure 6). We further analyzed the gene expressions by mining different database that contain soybean microarray data; however, owing to the less specificity of gene probe, data derived from microarray were not compatible with RNA-seq. However, RNA-seq provided notable discrimination within a certain gene family, particularly in inspecting genes that were weakly expressed but with high similarities. As qRT-PCR was an efficient validation method, we investigated their expressions in four major tissues, which further confirmed spatial expression patterns for CWI and VI genes (Figure 7a). Unfortunately, due to gene duplications and high identifies of cDNA within the CI subfamily, several CI gene expressions were not well evaluated by qRT-PCR (data not shown).

Among the serious biological threats in crops, fungi are considered to be one of the most dangerous pathogens to food security [53]. Activation of defense responses upon pathogen attack is accompanied by a rapid induction of the secondary and sugar metabolism-related genes [54]. As the common species isolated from soybean roots, *F. solani* is a fungal parasite that causes severe root rot and vascular wilt [55]. Besides, drought stress (water-deficit), as one of significant abiotic factors, has caused serious agricultural yield losses [56], prompting an urgent need to generate plants with optimized water use efficiency and high stress tolerance. As an essential phytohormone, ABA was discovered as a plant growth inhibitor in regulating developmental processes, including storage proteins and abiotic stress response [57]. In this study, based on search of cis-elements in promoter, we analyzed the gene expression patterns in response to stress factors. Our results showed that expression effects of CWIs were in a manner of more sensitive to the pathogenic stress than VIs, and transcripts of at least four *GmCWIs* were significantly induced in roots during the period fungal infection, particularly for 72 h inoculation (Figure 7b), indicating the potential of CWI involvement in the regulation of extracellular sugar partitioning and fungi colonization. In agreement with a recent report, *GmCWI3* (Gly.15g024600) demonstrated strong up-regulation within the gene group that is associated with carbohydrate metabolism upon infection of pathogenic *F. oxysporum* [58]. In contrast to pathogenic stress, under water-deficit and ABA perturbation, a few CWI and VI genes were affected and their transcripts were promoted significantly after 120 h treatment. Besides, two functionally characterized invertase inhibitors, *GmCIF1* and *GmC/VIF2*, were found to be consecutively up-to-down regulated, and displaying similar expression patterns to some CWI genes under stressful conditions (Figure S4). However, more specifically, *GmC/VIF2* was co-expressed with neither of the CWI genes in root infected by *F. solani*, whereas both *GmCIF1* and *GmC/VIF2* appeared to be co-induced with three CWI genes in leaves in response to water-deficit and ABA perturbation (Figure 7b). Co-expressions between CWI genes, invertase inhibitors, and sugar transporters provide the potential for the physical interaction of their protein products. Such interaction would help to maintain sucrose homeostasis and efficient distribution of liberated hexoses to apoplastic sink tissues via the modulation of enzyme activities [59]. Nevertheless, as several CWI genes were characterized by co-expression analysis upon these stress factors, it remains to be determined which CWI gene(s) would be the predominant target(s) of the *GmCIF1* or *GmC/VIF2* under stress conditions.

### 3.3. Posttranslational Mechanism underlying Stress Responses

In apoplastic space, the external supply of sugars is much needed by sink tissues for the plant development, but during infection, the imported sugar is inclined to be transferred to fungal sink and used as essential carbon resources to facilitate the fungi colonization [60]. The interaction between fungi and the hosts rely much on the sugar uptake and partition toward the specialized membranes of apoplast at plant-fungal interfaces [7]. Rapid increases in CWI expression and activities upon pathogenic fungal infection have been reported in different plant species [60,61,62,63], suggesting CWI as an essential activator in plant immune responses. Our results support this notion via the findings that several *GmCWIs* in roots were enhanced at 48 h after inoculation of *F. solani* (Figure 7b), prompting the significance of CWI as defense signals upon pathogen colonization. When comparing CWI proteins and activities with soluble sugar accumulation, it was noticed that CWI activities showed a trend of steady increase during the whole infective process and concurrent with elevated production of glucose and sucrose. This phenomenon was in line with the observation of the increased CWI proteins and activities (72 h), indicating that the contribution of CWI to glucose release in apoplast was especially limited to a later phase of fungi infection (Figure 8g). Our result was corroborated by a recent study, showing that suppression of CWI by inhibitory proteins *in planta* led to an improved susceptibility of plant to *Pseudomonas syringae* pv. tomato DC3000 [64]. We hypothesized that the accumulated glucose might be due to the consequence of depressed transcripts (activities) of *GmC/VIF2*, which in turn raised CWI activities. However, what specific factors in host or pathogen involved in suppression of *GmC/VIF2* remains to be envisioned. Fungal pathogen modified the host metabolism resulted in an energy increase and the production of carbon sources. CWI activities triggered a plant immune response that led to fine-tuned hexose balance in apoplast during the early phase of infection [60,65]. Thereby, it is plausible that the slightly increased glucose could act as a strong signaling molecule to negatively impact on *GmC/VIF2* expression as well as the elevation of CWI activities. Moreover, inhibitor-dependent posttranslational derepression of CWI may function in balance sucrose/hexoses ratio within the apoplastic space, which triggered sugar-mediated sweet defense [32]. Similarly, the pre-treatment of sucrose drastically reduced the symptoms of fungal infection in rice plant [66]. Elevation of CWI activities was essential for sugar uptake and distribution to benefit the pathogen development [62]. Even though, more recent reports showed that the innate invertase of pathogen might exert a functional role to accelerate sucrose hydrolysis by boosting the invertase activity in apoplast, suggesting a large proportion of contribution of pathogenic invertase to the maintenance of the sugar demand during infection [14,67].

The complex regulatory processes of plant drought tolerance involved the control of water influx and cellular osmotic adjustment [57]. Under water-deficit conditions, the accumulation of storage carbohydrate in wheat was in correlation with acid invertase gene expressions [68]. In our study, although a number of CWI and VI genes were significantly promoted, gene expressions appeared to increase in parallel with reductions of acid invertase activities and glucose content (Figure 8h). These results showed the inconformity between mRNA and enzyme activities (proteins). Thus, we postulated that the modulation of acid invertase, particularly CWI, may be largely regulated by their inhibitors, which showed constant increases of their expressions (Figure S4). As a consequence, sucrose metabolism might be decelerated and less hexose were liberated into apoplastic spaces. Likewise, a recent report revealed that posttranslational mechanisms led to a fine-tuned extracellular invertase to improve drought tolerance in crops [69]. There is also a plenty of evidence that transcript levels often change to a much greater extent than protein levels, and with different time courses [70]. Thus, invertase activities rather than gene expressions was considered to be a more direct and specific indicator of the plant performance and stress responses under drought conditions [69]. Besides, upon ABA treatment, transcript analyses upon ABA perturbation suggested that the two inhibitors are both ABA-responsive genes (Figure S4). Again, we found that the increases of gene transcripts were not able to elevate their enzyme activities, indicating the occurrence of posttranslational mechanisms. Likely, ABA promoted CWI encoding genes concomitant with two invertase inhibitors; however, CWI activities were subsequently suppressed by specific target of inhibitors at posttranslational level. These data fit well with recent studies that under the ABA stress regime, the posttranslational elevation of CWI activities resulted in a delay of leaf ageing, and invertase inhibitors might function as the essential components in the ABA-triggered leaf senescence [15,28]. Even though, other potential factors could be responsible for the regulation of CWI and VI at the posttranslational level. The protein degradation rate may increase under stress conditions, leading to the net loss of invertase proteins. These factors included CI and sugar transporters that could alter hexoses pool in vacuoles in plants and they may give rise to side impact on the homeostasis of sucrose metabolism within the apoplast. Nevertheless, information of biological activities of proteins provided here is vital to implicate how genes affect phenotypes. As lacking patterns of gene expression, protein and enzyme activity, posttranslational regulation of CWI or VI has received less attention. Here, examination of transcript fluctuation of genes and enzyme activities contributes to future in-depth understanding of posttranslational mechanisms underlying the molecular interaction of acid invertase and native inhibitors and to improve plant stress tolerance.

### 3.4. Potential Roles of GmCWI4

Extensive overview of genomic and expression patterns of invertase candidates in soybean allows for us to further identify their functional roles in vitro and in vivo. Within the AI gene family, the analysis of phylogenies and motifs revealed that GmCWI4 was clustered in the CWI subfamily and featured well conserved protein structures, showing 63% homology with AtCWI1 and 75% homology with VfCWINV2 (Figure 5). Besides, *GmCWI4* was particularly expressed in roots and leaves. Transcript of *GmCWI4* was drastically induced in response to stresses of fungi pathogen and water-deficit (Figure 7b). Based on molecular information above, we hypothesized that GmCWI4 may behave as an extracellular invertase in vitro. Plant species are likely to contain various types of invertase isoforms, which are directed to go into intracellular or extracellular compartments by protein targeting elements. For GmCWI4, the putative signal peptide was predicted to ultimately secret a mature protein to apoplast (Figure S1). This was well confirmed with the subcellular detection that *GmCWI4-YFP/RFP* fusion proteins, were observed primarily in the cell wall by using both transient transformation and stable transformation in onion epidermis and Arabidopsis root tip, respectively (Figure 9a,b), suggesting a potential feature of the apoplastic protein. However, the further evaluation of subcellular target of GmCWI4 in native plant remains to be conducted.

Previously, two Arabidopsis CWIs were originally annotated as extracellular invertase; however, they were verified as lacking sucrose hydrolysis activities and appeared to be involved in the fructan degradation [71]. Two rice VI genes were unexpectedly characterized with FEH activities but actually no fructan biosynthesis genes were identified in rice genome [72]. In addition, in vitro analysis of tobacco CWI resulted in findings of the defect invertase, Nin88, which failed to hydrolyze sucrose [73]. FEHs lack in invertase activity that appear to be derived from cell wall invertase-type ancestors by a few rounds of evolution events. Based on an assay of the site-directed mutagenesis, an Asp-239 homolog is experimentally verified as a key determinant to distinguish genuine CWIs from defect CWI/FEHs [74]. Nevertheless, heterologous expression of *GmCWI4* in tobacco leaves significantly increased CWI activities, but not VI activities, indicating its functional role as CWI, which are consistent with the functional assay by using the recombinant enzyme purified from *Pichia pastoris*, showing the preferential hydrolysis of sucrose substrate, but no detected FEH activity (Figure 9c–e). Overall, extracellular invertase plays critical roles in the regulation of various physiological processes upon stress cues. The advantage of functional specificity of a cell wall-targeted GmCWI4 demonstrated here is a valuable candidate for engineering to enhance plant stress adaptation. Our work opens a new avenue that may pave the way for maintaining sucrose metabolism and defense regulation within source-sink dynamics.

## 4. Materials and Methods

### 4.1. Plant Materials and Growth Conditions

*Glycine max* cultivar, Heinong 53 from Soybean Research Institute (Harbin, China) was grown in standard potting soil in the greenhouse, the temperature was set at 18 °C (8 h light) and 24 °C (16 h dark), with a relative humidity of 55%, and light intensity of 350–400 μE [29]. Open flowers were sampled in parallel with leaf tissue. Pod and seeds were harvested by seed weight and pod lengths correlating to approximate days after flowering (DAF), root and nodule were harvest, as specified [50]. *Arabidopsis thaliana* (ecotype *Col-0*) was grown in the greenhouse or on agar medium in growth chambers at 22 °C, and relative humidity of 55% under long day (16 h light/8 h dark, 100–150 μE) conditions. Tobacco (*Nicotiana benthamiana*) plants were cultured in a green house at 25 °C under a light regime of 16 h and 300 μE. All of the harvests consisted of samples that were pooled from a minimum of three plants that were taken at the same time of a day.

### 4.2. Plant Transformation

Agrobacterium-mediated transformation in Arabidopsis by floral dip and transient expression in tobacco by infiltration were performed, as described previously [28]. To visualize the subcellular localization of GmCWI4 in onion epidermal cells, a transient expression system was established based on an optimized protocol. Control plasmid (SecGFP) and YFP-fusion plasmid were bombarded with 1.6 μm gold particles while using the Model PDS-1000/He Biolistic Particle Delivery System (Bio-Rad, Berkeley, CA, USA) with 4481 kPa helium pressure, a vacuum of 86 kPa and a distance of 6 cm. Bombarded cells were incubated for 48 h and then visualized by confocal laser scanning microscopy (CLSM) (Zeiss, Germany). GFP fluorescence detection of transformed cells should treat with 20 mm PIPES buffer (pH 7.0) for 3 h after 48h incubation.

### 4.3. Stress Treatments

Inoculum of *F. solani* was initially grown on potato dextrose agar (PDA) medium at 25 °C in the dark for seven days. Afterwards, fungal spores were suspended with sterile distilled water by scraping the surface of agar cultures. The concentration of fungal spores was adjusted to 2.0 × 10^6^ spores per milliliter via calibration of a fungal spore suspension from a fungal colony of agar plate. Plants at 21 days after germination (DAG) were inoculated with 20 mL spore suspensions into the peripheral areas of roots for 48 h and 72 h. Plants with fully expanded trifoliate leaves (28 DAG) were used to induce water-deficit stress after water was withheld for 48 h and 120 h. Roots and trifoliate leaves upon *F. solani* and water-deficit treatment were harvested for preparations of RNA and protein extraction, respectively. For ABA treatment, fully expanded trifoliate leaves (28 DAG) were sprayed with 100 μM ABA (dissolved in 10% ethanol) once a day for 5 days. After growing for 2 days (48 h) and 5 days (120 h), mature leaves were harvested for RNA isolation and the determination of acid invertase activities.

### 4.4. Genomic Analysis and Phylogenetic Tree

Based on known invertase genes in Arabidopsis and rice, a homologous BLAST was performed to identify soybean invertase genes in soybean genomic sequence (Wm82.a2.v1) within Soybase (https://www.soybase.org/) (14 December 2009). The protein sequences of the members within specific gene subfamilies were aligned while using the MUSCLE algorithm of the Molecular Evolutionary Genetics Analysis (MEGA) 6.0 software (http://www.megasoftware.net/) (16 October 2013) [43]. A phylogenetic tree was constructed using neighbor-joining method from protein sequences of 90 members (Figure 5). Statistical support was given as consensus bootstrap values from 1000 replicate tests for each tree. The phylogenetic trees are drawn to scale, with branch lengths in the same units as those of the evolutionary distances that are used to infer the phylogenetic tree.

### 4.5. Expression Analysis

The transcriptome sequencing (RNA-seq) of tissue-specific expressions of invertase genes was performed, as described previously [50]. Gene expression levels were estimated using the values of number of mapped reads per kilobase of the exon region per million mapped reads (RPKM). Data contained within the RNA-seq is obtained from the public Soybean eFP Browser (http://bar.utoronto.ca/efpsoybean/cgi-bin/efpWeb.cgi) (17 December 2017). RNA extraction and cDNA synthesis for qRT-PCR were performed, as described previously [75]. The selected tissues were homogenized under liquid nitrogen using a Tissue Lyser (Qiagen, Hilden, Germany). Total RNA was extracted by using the Gene MATRIX Universal RNA purification Kit (Qiagen), according to the manufacturer’s instructions. RNA quality was determined by NanoDrop One (Thermo, Whitby, ON, Canada). Reactions were run on a CFX96 Touch qPCR Systems (Bio-Rad). Primer efficiency was tested in preliminary experiments with series dilutions of cDNA, producing an R2 value ≥0.99. The relative expression level of a target gene was calculated by normalizing to the geometric mean of multiple reference genes, and values of the control (0 h) were set up at 1. Primer sequences were listed in Table S2. Each experiment was conducted with three biological repeats, each sample with three technical replicates. Heatmaps were constructed with the CIMminer online program (http://discover.nci.nih.gov/cimminer/home.do) (19 July 2018).

### 4.6. Sequence Isolation and Subcellular Localization

Coding sequences (stop codon removed) of *GmCWI4* were amplified by PCR using primers containing the Gateway *attB1* and *attB2* recombinant sites (Table S2). The amplified fragments were recovered by using the QIAquick PCR purification kit (Qiagen, Germany) and then inserted into *pDONR201* donor vector. After sequencing, the donor vector subsequently recombined with the destination vector *pK7RWG2.0*, yielding the GmCWI4-RFP constructs. Onion epidermis was bombarded with *A. tumefaciens* (*C58C1*) harboring *pK7RWG-GmCWI4* and a cell wall-localization marker *pMDC32-SecGFP*. Additional YFP fusion construct, *pB7YWG-GmCWI4* was introduced into Arabidopsis plants that were transformed with membrane marker (*pK7RWG-AtRLP44*). Transfected regions of onion epidermis and root tip of four days transgenic Arabidopsis were analyzed by a LSM 510 Meta inverted microscope (Zeiss, Jena, Germany). The yellow fluorescent protein (YFP) was excited with a 514-nm laser line, and the emitted fluorescence was captured while using a 530-600 nm band pass filter. The red fluorescent protein (RFP) was excited with 543-nm laser line and the emitted fluorescence was collected with a 560-nm long pass filter.

### 4.7. Heterologous Expression and Purification of GmCWI4

The coding sequence (CDS) of GmCWI4 was amplified by PCR and cloned into *pMDC32*. The overexpressing construct was transiently introduced into tobacco epidermal cells by infiltration. After 40 h, infiltrated leaves were harvested for determination of extracted invertase activities. For recombinant protein overexpression in yeast *Pichia pastoris* (*X-33*), CDS of *GmCWI4* lacking signal peptide (51 nt) was amplified by PCR and cloned into *pPICZαA* vector using *EcoRI* and *XbaI* restriction sites, containing a secretory signal of α-factor in N-terminus. Preculture of 25 mL BMGY medium with addition of 100 μg/mL Zeocin was inoculated with single colonies of transformants, which were screened for the efficient protein expression. 700 mL BMGY medium was inoculated with 10 mL preculture in baffled Erlenmeyer flasks (2 L). After incubation for 72 h at 30 °C and 220 rpm, protein expression was induced by medium change to methanol-containing BMMY medium and was incubated for 72 h at 30 °C and 220 rpm. The protein containing culture medium was harvested by centrifugation and the supernatant was precipitated with 80% ammonium sulfate by stirring for 1–3 h at 4 °C, and subsequent centrifugation for 1–3 h at 15,000 × *g* and 4 °C. Precipitated proteins were dissolved in 5 mL LEW buffer (50 mM sodium phosphate buffer, pH 7.5, 500 mM NaCl, and 10% glycerol) and dialyzed overnight against LEW buffer. Recombinant proteins were purified via His6-tag Ni^2+^ affinity purification over a column packed with 500 mg Ni-IDA (Macherey-Nagel) according to the manufacturer’s instructions and eluted in 5 × 1.5 mL fractions with elution buffer (50 mM sodium phosphate buffer, pH 7.5, 500 mM NaCl, 10% glycerol, 250 mM imidazole).

### 4.8. Determination of Invertase Activities

Specified tissues (100 mg) were sampled and ground separately in liquid nitrogen for the enzyme extraction. The in vitro determination of acid invertase activities of specific tissues were performed, as described previously [76]. Preparations (100 μL) were mixed with 100 μL sucrose (100 mM) up to 300 μL and incubated for 1 h at 37 °C. The reaction was terminated by 30 μL sodium phosphate (1 M, pH 7.5), and incubated for 5 min at 95 °C. The assay was conducted in quadruplicate, one of which was neutralized and boiled immediately after the addition of sucrose. Sample activity was subtracted from the activity of the others as background absorption. Liberated glucose was measured in a coupled enzymatic-optical assay. In this assay, 100 μL of the reaction, 20 μL 30 mM ATP, 20 μL 30 mM NADP, and 2 μL suspension (340 U/mL HK, 170 U/mL G6P-DH, Roche) mixed up to 1 mL with buffer (40 mM triethanolamine, 8 mM MgSO_4_, pH 7.5) and incubated for 5 min at room temperature. Synthesis of NADPH was detected by U-2000 UV/VIS spectrophotometer (Hitachi, Japan) at 340 nm, and finally, the released glucose was calculated while using the Lambert-Beer law. Invertase activity was defined as expression in nkat/g fresh weight (1 nkat=1 nmole Glcose liberated/second).

### 4.9. Immunoblotting

After electrophoresis with equal loaded samples, proteins were transferred to a polyvinylidene difluoride (PVDF, Bio-Rad) membrane in a Novex^TM^ semi-dry blotting device (Thermo, Canada). Immunodetection was conducted with polyclonal antisera (generated from sugar beet BvCWI-1 and BvVI1) raised against extracted CWI and VI fractions of roots and leaves. An anti-rabbit inmunoglobulin horseradish peroxidase (HRP) conjugate was used as a secondary antibody. Immunosignals were detected using the SuperSignal West Dura Extended Duration Substrate kit (Thermo, Canada). After film exposure, membranes were stained with amido black (0.1% amido black, 45% ethanol, 10% acetic acid) to analyze the loaded protein. Antiseras were used in a dilution of 1:10,000 or 1:20,000. A commercially available c-MYC antibody was used for the western blotting in a dilution of 1:500.

### 4.10. Functional Assay and Carbohydrate Quantification

For the invertase characterization, concentrated culture medium was assayed in triplicate. Sucrose was used as substrate for detecting invertase activity. 50 µL of protein extract was incubated with 50 µL of substrate (4 mg/mL) solution for 1 h at 30 °C. Reactions were stopped by inactivating enzymes through boiling for 5 min at 95 °C. The reaction products were identified and quantified via anion exchange chromatography with pulsed amperometric detection (HPAEC-PAD) with ICS-3000 system on a Dionex^TM^ Carbopac PA1 analytical column (Thermo, Canada). Specific activities were calculated as nmol product produced per mg total protein per min. Soluble sugars (glucose, fructose and sucrose) were quantified similarly, as described above.

## 5. Conclusions

Systematic survey of gene structure and expression profiles in sugar metabolism is vital to modern plant biology, in aspect of engineering of crops for desirable traits. In the present work, we performed extensive sequence analyses and provided detailed genomic information of 32 identified invertase candidates in soybean. Transcript analyses of invertase genes showed their spatiotemporal expression patterns in response to stress stimuli. Detection of proteins, enzyme activities, and soluble sugar content suggested that the modulation of CWI might depend on posttranslational mechanisms, which was integrated much by inhibitory proteins. Further functional characterization of a novel extracellular invertase in vitro offers indispensable information in understanding of its regulatory roles and molecular mechanisms in planta under stressful regime. In conclusion, initial findings that are presented here prompted us to enhance plant performance in legume agriculture via the technology advances. As specific CWIs were highly regulated in response to stress cues, future work will attempt to explore their physiological significance by analyzing the possible phenotypes of mutants and molecular interaction with inhibitors under the stress exposure.

## Figures and Tables

**Table 1:** Numbers of identified invertase genes within different plant species.

Species	CWI	VI	CI	References
	Numbers of the Different Invertase Sub-Families			
*Arabidopsis thaliana*	6	2	9	[34]
*Oryza sativa*	8	2	8	[35]
*Malus domestica*	3	3	12	[36]
*Manihot esculenta*	6	3	11	[37]
*Populus trichocarpa*	5	3	16	[38]
*Vitis vinifera*	1	2	3	[39]
*Solanum lycopesicum*	4	2	7	[6]
*Camellia sinensis*	3	3	8	[40]
*Saccharum* hybrids	7	1	6	[41]

## Supplementary Materials

Supplementary materials can be found at http://www.mdpi.com/1422-0067/19/8/2395/s1. Figure S1: Multiple sequence alignment of the acid invertase (CWI and VI) gene family in soybean, Figure S2: Multiple sequence alignment of the cytoplasmic invertase (CI) family in soybean, Figure S3: An unrooted phylogenetic tree of the soybean invertase family, Figure S4: Expression detection of inhibitor genes in response to stress factors, Figure S5: Extracted acid invertase activities in various soybean tissues, Figure S6: Recombinant protein purification and detection of the enzyme activities, Table S1: Lists of the invertase gene candidates in soybean, Table S2: The lists of primers were used for qRT-PCR, subcellular localization and protein expression.

## Abbreviations

CWI	Cell Wall Invertase
VI	Vacuolar Invertase
AI	Acid Invertase
CI	Cytosolic Invertase
Susy	Sucrose Synthase
FEH	Fructan Exohydrolase
UDP	Uridine Diphosphate
GH32/100	Glycoside Hydrolase family 32/100
ROS	Reactive Oxygen Species
