# Supplementary material for: Genome-Wide Survey of Invertase Encoding Genes and Functional Characterization of an Extracellular Fungal Pathogen-Responsive Invertase in Glycine max
Source: Int J Mol Sci. 2018 Aug 14;19(8):2395. doi: 10.3390/ijms19082395 (PMC6121457; doi:10.3390/ijms19082395)
Supplement: Supplementary file 1 [file ijms-19-02395-s001.pdf]

Table S1. Lists of the invertase gene candidates in soybean.

Gene	Gmax 2.0	fl cDNA NCBI	Chr	Location	Gene Model	ORF (bp)	MW (kDa)	pI	Target
GmCWI1	Glyma.07G008800	NM_001249396.1	Chr07:	647865-652858	1	1695	63.54	8.84	Apoplast
GmCWI2	Glyma.08G192000	XM_003531563.3	Chr08	15440745-15445678	1	1695	63.60	9.22	Apoplast
GmCWI3	Glyma.15G024600	XM_003546647.3	Chr15	1992572-1996208	1	1728	64.39	8.75	Apoplast
GmCWI4	Glyma.13G349300	XM_003543542.3	Chr13	43903578-43906955	2	1725	64.45	9.16	Apoplast
GmCWI5	Glyma.08G191900	n.a	Chr08	15431365-15437778	1	1545	61.37	8.76	Apoplast
GmCWI6	Glyma.14G096600	XM_003544491.3	Chr14	9045982-9049929	3	1713	65.09	5.87	Apoplast
GmCWI7	Glyma.10G074800	XM_003535691.3	Chr10	7745865-7752555	1	1731	65.43	9.48	Apoplast
GmCWI8	Glyma.17G227800	XM_006601164.2	Chr17	38275485-38278808	2	1689	64.41	5.08	Per
GmCWI9	Glyma.17G227900	XM_003549299.3	Chr17	38284476-38287851	2	1659	63.25	5.17	Per
GmCWI10	Glyma.20G029100	XM_003556688.3	Chr20	3460364-3464727	2	1668	62.90	8.42	PM
GmCWI11	Glyma.19G195400	XM_003553553.3	Chr19	45262941-45267834	1	1665	64.78	9.10	Apoplast
GmCWI12	Glyma.03G197400	n.a	Chr03	40681218-40685449	1	1605	60.40	8.39	Apoplast
GmVI1	Glyma.01G211000	XM_006573669.2	Chr01	54261857-54266740	1	2028	75.06	5.43	Chl
GmVI2	Glyma.05G056300	XM_003525755.3	Chr05	5148962-5154038	3	1938	71.76	5.66	PM
GmVI3	Glyma.06G318500	XM_003526326.3	Chr06	50710931-50716437	2	1941	72.13	5.36	PM
GmVI4	Glyma.09G231500	XM_003533466.3	Chr09	45478768-45483760	2	1893	70.59	5.39	PM
GmVI5	Glyma.11G030800	XM_003538679.3	Chr11	2225720-2230176	1	1860	68.61	5.10	Per
GmVI6	Glyma.12G005100	XM_003540527.3	Chr12	395447-400775	2	1845	68.86	5.70	PM
GmVI7	Glyma.17G138500	XM_003549854.3	Chr17	11222613-11227294	2	1938	71.98	5.18	PM
GmCI1	Glyma.03G230400	XM_003520741.2	Chr03:	43205651-43209277	1	1992	75.25	5.47	ER/Cyto
GmCI2	Glyma.04G005700	XM_003523456.3	Chr04:	460114-463655	5	1713	65.24	6.78	Per/PM
GmCI3	Glyma.05G185500	XM_014775774.1	Chr05:	37243691-37249494	4	1959	74.10	6.39	Mit/PM
GmCI4	Glyma.06G005400	XM_006581041.2	Chr06	446722-450595	2	1722	65.39	6.34	Per/PM
GmCI5	Glyma.07G236000	XM_003529455.3	Chr07:	41783573-41788799	1	2040	77.00	6.13	Chl/PM
GmCI6	Glyma.08G143500	XM_003531340.3	Chr08:	10949673-10956219	1	1959	73.87	5.84	Mit/PM
GmCI7	Glyma.10G145600	XM_003535267.3	Chr10:	38035440-38039395	1	1956	73.73	6.59	Chl
GmCI8	Glyma.10G214700	XM_014763320.1	Chr10:	44674211-44679453	4	1668	63.21	6.11	PM
GmCI9	Glyma.12G024000	XM_014764424.1	Chr12:	1748002-1751739	3	1674	63.58	6.02	PM
GmCI10	Glyma.17G037400	XM_003550769.3	Chr17:	2732048-2737399	2	2043	77.02	5.94	Chl
GmCI11	Glyma.19G227300	XM_006605197.2	Chr19:	47882570-47886680	1	2016	76.01	5.43	Cyto
GmCI12	Glyma.20G095200	XM_003555130.3	Chr20	33827363-33831352	1	1959	73.84	6.79	Cyto/Chl
GmCI13	Glyma.20G177200	XM_014772800.1	Chr20:	41446962-41451980	2	1668	63.25	6.22	Per/Chl

fl cDNA, full length complementary DNA; Chr, chromosome; ORF, open reading frame; MW, deduced molecular weight of the protein; pI, isoelectric point; Per, Peroxisome; PM, Plasma Membrane; Chl, Chloroplast; ER, Endoplasmic Reticulum; Cyto, Cytosol; Mit, Mitochondria; n.a, not applicable. The gene ID was obtained from the genome assembly nomenclature of Glyma.Wm82.a2 in Soybase database (https://soybase.org/). Predictions of protein subcellular targeting were performed by programs of PSORT (https://wolfpsort.hgc.jp/) and CELLO (http://cello.life.nctu.edu.tw/). Protein molecular weight (MW) and isoelectric point (pI) were deduced by the program of ExPASy (http://web.expasy.org/compute_pi/).

Table S2. The lists of primers were used for qRT-PCR, subcellular localization and protein expression.

qRT-PCR

Gene	Gene ID NCBI	Gene ID 2.0	Primers	Size
GmEF/αb	EV279336	Glyma.02G276600.1	F1: CCACTGCTGAAGAAGATGATGATG<br>R1: AAGGACAGAAGACTTGCCACTC	182 bp
GmCYP	CF806591	Glyma.12G024700.1	F1: ACGACGAAGACGGAGTGG<br>R1: CGACGACGACAGGCTTGG	121 bp
GmACTII	BW652479	Glyma.02G091900.1	F1: ATCTTGACTGAGCGTGGTTATTCC<br>R1: GCTGGTCCTGGCTGTCTCC	161 bp
GmACT2/7	BW677100	Glyma.04G215900.1	F1: CTTCCCTCAGCACCTTCCAA<br>R1: GGTCCAGCTTTTCACACTCCAT	198 bp
GmCWI1	Glyma07g01090	Glyma.07G008800.1	F1: TTGACTTCTTCTACTGATGCCTCT<br>R1: ACGTTGGTGGCAAGTGTCAG	162 bp
GmCWI2	Glyma08g20490	Glyma.08G192000.1	F1: AGCAATTGCCATGACTATGTCTA<br>R1: GGGAGGTTGGAAGTGATAAGCA	157 bp
GmCWI3	Glyma15g02850	Glyma.15G024600.1	F1: ACCATAGTGCTTGTTATTCAGGT<br>R1: ACTGAGTTAAAATTGTTCCTGCC	182 bp
GmCWI4	Glyma13g42530	Glyma.13G349300.1	F1: AGTGCCTGCAAATTTCATTACTGT<br>R1: GTTGACAGGCCTCTAAAACCT	121 bp
GmCWI5	Glyma08g20480	Glyma.08G191900.1	F1: ACTGCTTATCATTTTCAACCAGC<br>R1: AGTGGGGTCCAGTTCACAAG	161 bp
GmCWI6	Glyma14g11000	Glyma.14G096600.1	F1: ACAGAATTGGATGAATGGGCCT<br>R1: GAGCCTGACCAGCAGCTATT	198 bp
GmCWI7	Glyma10g08670	Glyma.10G074800.1	F1: TGGATACCATTTTCAACCTCGTAA<br>R1: TCCTTTGATACTGCGTGTCCC	147 bp
GmCWI8	Glyma17g34570	Glyma.17G227800.1	F1: TATCACTGCATCACAGGCCG<br>R1: CTGCAGTGTGTTCTGTTTGGT	191 bp
GmCWI9	Glyma17g34590	Glyma.17G227900.1	F1: TGAGTTTGTCCTTTGCGTGT<br>R1: CACCTACCACTCTGAGAAAAATGC	173 bp
GmCWI10	Glyma20g03620	Glyma.20G029100.1	F1: TGATACTATGAGCATCCCCTACAT<br>R1: GGAGAAGTTGACCGAGGCTT	166 bp
GmCWI11	Glyma19g38160	Glyma.19G195400.1	F1: ACTGGATCAACGGCTCAGTG<br>R1: CTATGGTGGCTGACCCAGAC	147 bp
GmCWI12	Glyma03g35520	Glyma.03G197400.1	F1: GCAAGCCAAACATCCGATCC<br>R1: GACGTCTCCAACCCTGCATT	102 bp
GmVI1	Glyma01g41990	Glyma.01G211000.1	F1: TAAGGGATGAGGGACTCGCA<br>R1: ATGACTCCCATTCCAGCAGC	152 bp
GmVI2	Glyma05g04290	Glyma.05G056300.1	F1: TCTGCCAAAGTGCCAAGTCA<br>R1: AGAAGGTCCTTGCGAGTGTG	188 bp
GmVI3	Glyma06g47640	Glyma.06G318500.1	F1: GGGTCGCAATTGGGTCAAAG<br>R1:TACCCGGAACCGCATGTAAG	111 bp
GmVI4	Glyma09g36580	Glyma.09G231500.1	F1: ACTGGATGAACGGTCCATTG<br>R1: GCTGACCCTGACCATACACC	193 bp
GmVI5	Glyma11g03360	Glyma.11G030800.1	F1: GGATGAACGGTCCAATGTTCT<br>R1: ACAGCGTGTCCCCAAACTAT	100 bp
GmVI6	Glyma12g00780	Glyma.12G005100.1	F1: CCTGACAATTGTTTCTCCAGCG<br>R1: TCCAAAGAAAGCTGAGTGCCA	169 bp
GmVI7	Glyma17g14750	Glyma.17G138500.1	F1:TAAAATGTGCCAATGTGAGCC<br>R1:GTTATGACTTGGCCCCAACTTT	114 bp

Co-localization

Gene	Primers
GmCWI4-pK7RWG/pB7YWG	CWI4-attB1: GGGGACAAGTTTGTACAAAAAAGCAGGCTCCATGGCCATATCTCCAA<br>CWI4-attB2-: GGGGACCACTTTGTACAAGAAAGCTGGGTGTTGATTTTTGCCTTC
attB1	attB1_adapter:GGGGACAAGTTTGTACAAAAAAGCAGGCT
attB2	attB2_adapter:GGGGACCACTTTGTACAAGAAAGCTGGGT
pDONR201	SeqLA: TCGCGTTAACGCTAGCATGGATCTC<br>SeqLB: GTAACATCAGAGATTTTGAGACAC

Pichia expression

Gene	Primers
GmCWI4-pMDC32	CWI4-attB1: GGGGACAAGTTTGTACAAAAAAGCAGGCTCCATGGCCATATCTCCAA<br>CWI4-attB2+:<br>GGGGACCACTTTGTACAAGAAAGCTGGCTCGTTCAGTTGATTTTTGCCTTCTTC
GmCWI4-pPICZαA	CWI4pPICZ-F1: AATCCGGAATTCTCTCAATGGTGTTCTTCCCATTG<br>CWI4pPICZ-R1: GACTAGTCTAGACCTCAGTTGATTTTTGCCTTCTTC
pPICZαA	5' AOX1:GACTGGTTCCAATTGACAAGC<br>3' AOX1:GCAAATGGCATTCTGACATCC
pDONRZeo	M13-fw: GTAAAACGACGGCCAGT<br>M13-rev: GGAAACAGCTATGACCATG

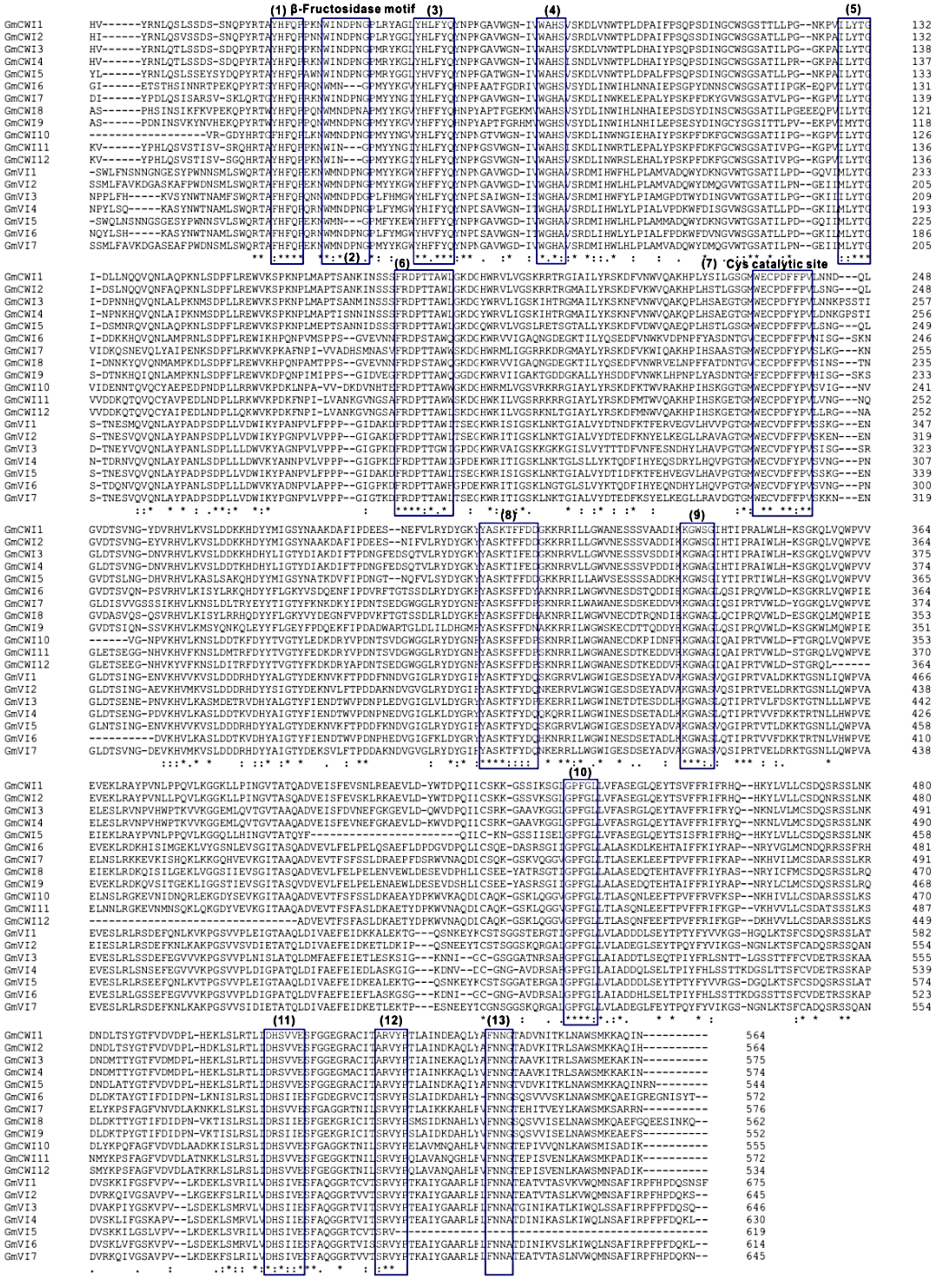

Figure S1. Multiple sequence alignment of the acid invertase (CWI and VI) gene family in soybean. The boxed region indicates the 13 well-conserved regions including typical β-Fructosidase motif and catalytic site from the known CWI and VI of the selected green plants.

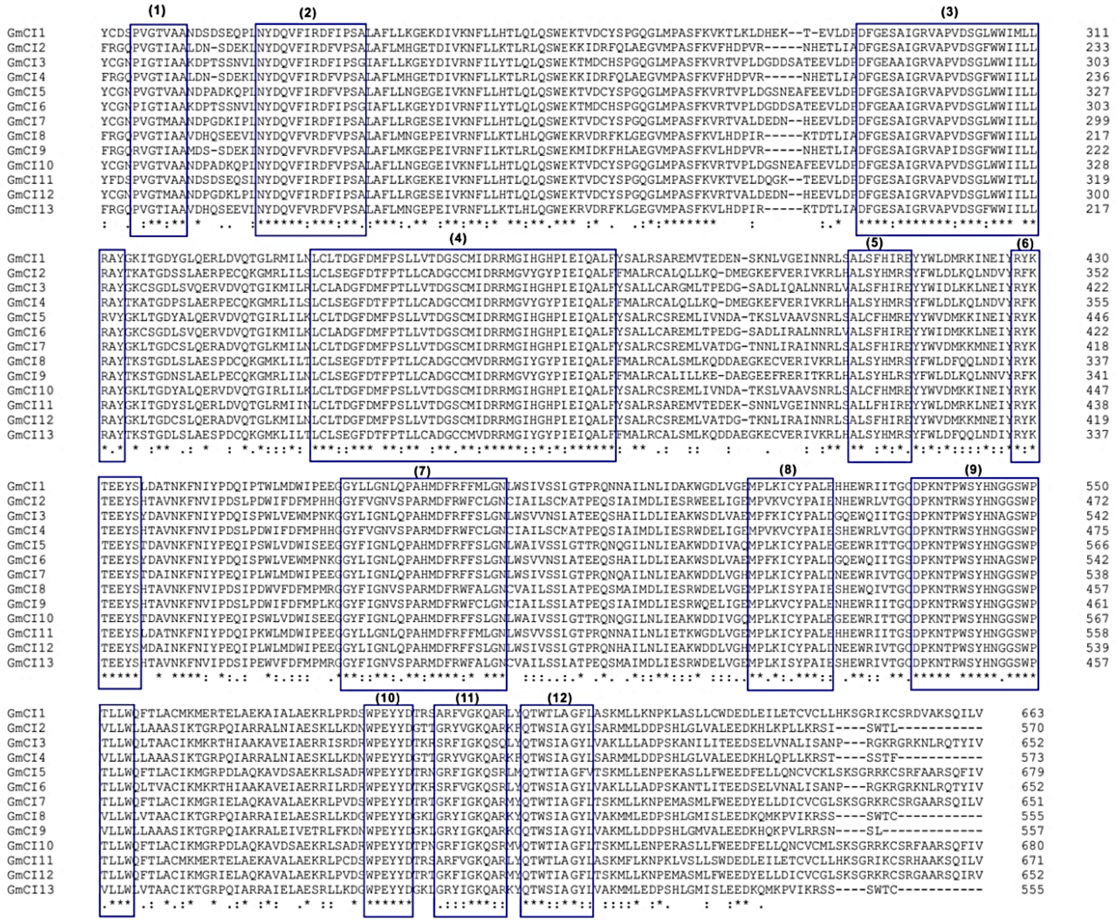

Figure S2. Multiple sequence alignment of the cytoplasmic invertase (CI) family in soybean. The boxed regions indicate the 12 well-conserved regions from the known CI of the selected green plants.

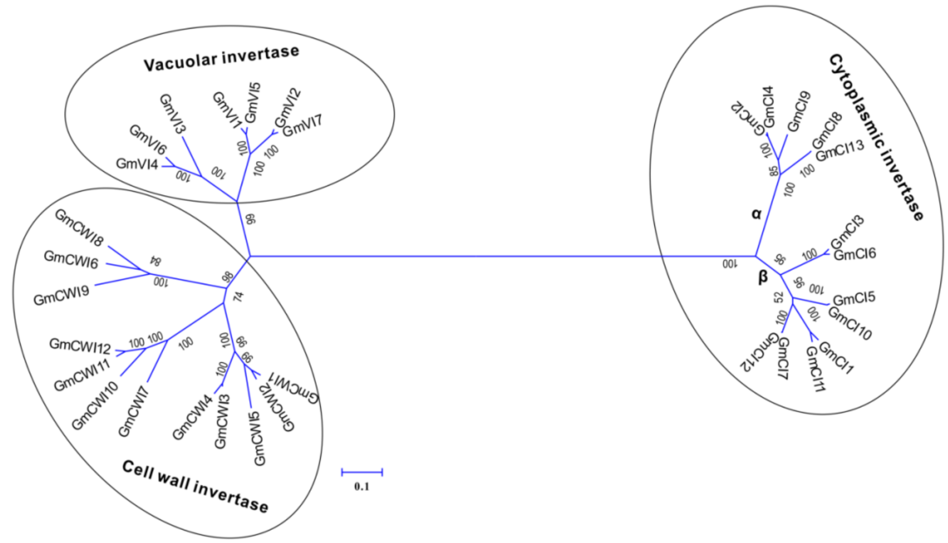

Figure S3. An unrooted phylogenetic tree of the soybean invertase family. The invertase family are divided into three subgroups. Phylogenetic analysis was done using neighbor-joining method in MEGA 6. The posterior probabilities have been multiplied by 100. The scale bar shows expected number of nucleotide substitutions per site.

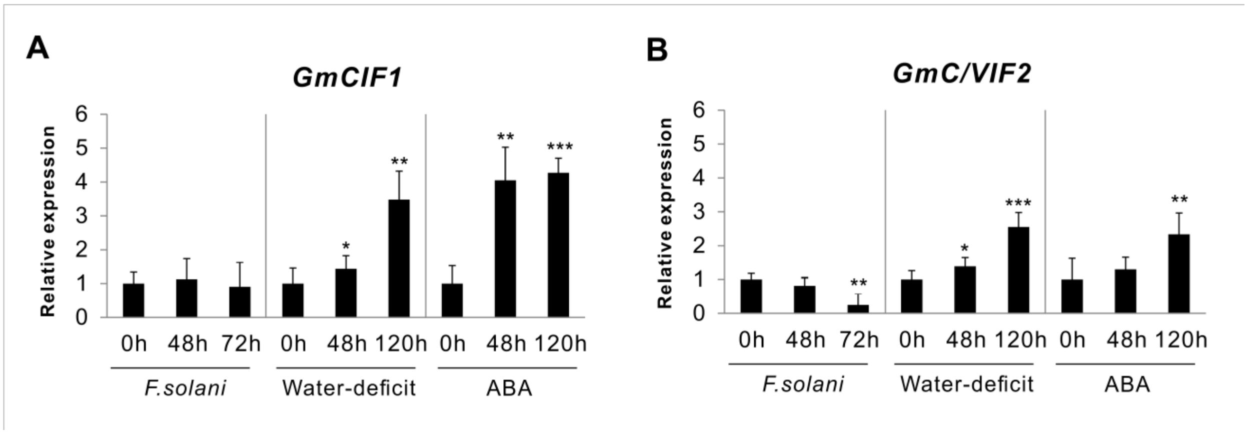

Figure S4. Expression detection of inhibitor genes in response to stress factors. Expressions were analyzed by qRT-PCR. Data represent mean values ±SE of at least three independent biological replicates. *GmACT2/7*, *GmACTII*, *GmEF/αb*, and *GmCYP* were used as reference genes. Asterisks indicate significant differences in comparison with the control using Student's t-test: ****P<0.001*, ***P<0.01*, **P<0.05*.

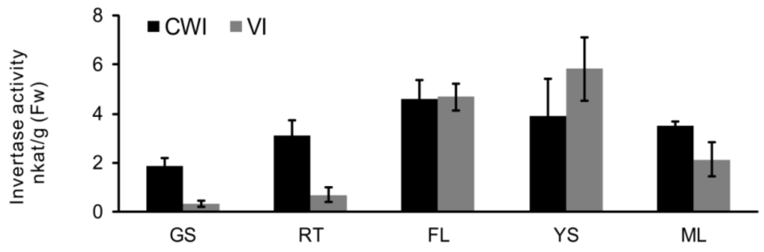

Figure S5. Extracted acid invertase activities in various soybean tissues. GS, 24 hours germinating seed; RT, root; FL, flower; YS, young seed; ML, mature leave. The enzyme activity data represent means ±SE of at least four independent biological replicates.

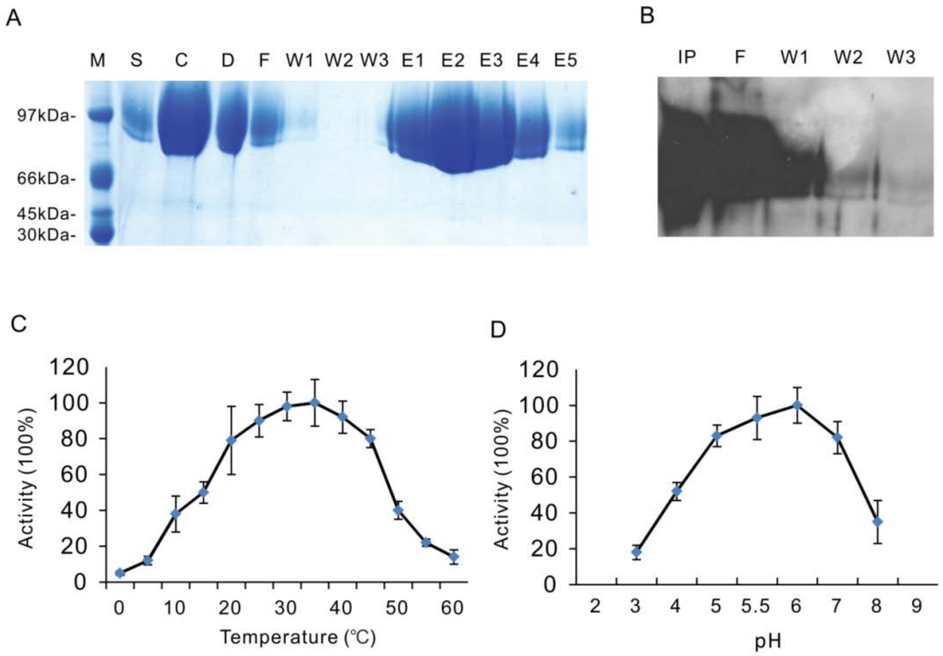

Figure S6. Recombinant protein purification and detection of the enzyme activities. Recombinant GmCWI4 from *Pichia pastoris* were induced and purified with culture supernatants via immobilized metal ion affinity chromatography on Ni-IDA resin, and analyzed by SDS-PAGE and Coomassie staining (a). Immunoblot analysis with a polyclonal antibody (c-Myc) was raised against a GmCWI4 protein fragment (b). Detection of enzyme activities under different incubation conditions (c, d). Data are means of three replicates. M: protein ladder, S: culture supernatant, C: concentrated culture supernatant, D: concentrated culture supernatant after dialysis, FT: column flow through, W1-3: wash steps, E1-E6: elution fractions. C Covalent coupling of purified GmCWI4 to HiTrap NHS-activated HP 5-ml column. Input: as loading control a 1:50 dilution of the actual protein solution was used, FT: column flow through, W1-7: washing steps for column inactivation. Activity is expressed as a percentage of the maximum activity calculated by the amount of fructose and glucose released after incubation of 25μg of recombinant GmCWI4 with 50μl sucrose substrate.
